# Effectiveness and safety of Wendan decoction for post-stroke depression: A protocol for systematic review and meta-analysis

**DOI:** 10.1097/MD.0000000000028297

**Published:** 2021-12-23

**Authors:** Yonghui Hou, Wenen Pang, Jing Gao, Wei Si, Baile Ning, Wenbin Fu

**Affiliations:** a Shijiazhuang People's Hospital, Shijiazhuang, Hebei, China; b The First Affiliated Hospital of Henan University of Traditional Chinese Medicine, Zhengzhou, China; c The Second Affiliated Hospital of Guangzhou University of Chinese Medicine, Guangzhou, Guangdong, China.

**Keywords:** meta-analysis, post-stroke depression, protocol, Wendan decoction

## Abstract

**Background:**

Post-stroke depression (PSD) refers to a series of affective disorder syndromes that occur after stroke and are often accompanied by physical symptoms. PSD presents with low mood and lack of interest as the main characteristics along with the symptoms of stroke. The physical symptoms of PSD include sleep disorder, loss of appetite, and reluctance to communicate. Although Wendan decoction has been suggested to be effective in the treatment of PSD, there is no meta-analysis providing evidence for the usefulness of Wendan decoction for treating PSD.

**Methods:**

The following electronic databases will be searched: the Cochrane Library, PubMed, EMBASE, China National Knowledge Infrastructure, Wan Fang databases, Chinese Biomedical Literature Database, and China Science and Technology Journal Database. Each database will be searched from its inception to November 2021. Two independent researchers will conduct study selection, data extraction, and risk bias assessment. Any discrepancies will be resolved through consultation with a third researcher. If the included data are suitable, we will conduct a meta-analysis using RevMan v5.4 software.

**Results:**

In this systematic review, the effectiveness and safety of Wendan decoction in the treatment of PSD will be evaluated.

**Conclusion:**

The findings of this meta-analysis will provide evidence-based data for the application of Wendan decoction in the treatment of PSD.

**Ethics and dissemination:**

Individual patient data and privacy will not be involved in this research,so ethics approval is not required.

**INPLASY registration number:**

INPLASY2021110018

## 1 Introduction

Post-stroke depression (PSD) refers to a series of affective disorder syndromes that occur after stroke and are often accompanied by physical symptoms.^[1]^ PSD presents with low mood and lack of interest as the main characteristics, and the physical symptoms include sleep disorders, loss of appetite, and reluctance to speak.^[2]^ One study showed that the overall prevalence of PSD in China was 34.9% [95% confidence interval (95% CI): 31.7–38.1], and the prevalence of PSD in female patients (41.6%) was higher than that in male patients (32.0%).^[3]^ In addition to its negative effects on the outcomes of rehabilitation treatment, PSD is also known to increase the mortality rate and the risk of stroke recurrence, reduce patients’ quality of life and their social participation, and impose a heavy burden on the patient's family and society.^[4]^

Antidepressants such as selective serotonin reuptake inhibitors (SSRIs), serotonin-norepinephrine reuptake inhibitors (SNRIs), monoamine oxidase inhibitors (MAOIs), and tricyclic antidepressants (TCAs) have been prescribed to treat PSD. However, antidepressant treatment in patients with PSD is associated with the risk of adverse events, including anxiety-like episodes, lethargy or insomnia, restless leg syndrome, hyponatremia, nausea and vomiting, postural hypotension, erectile dysfunction, drug dependence.^[5,6]^ Moreover, a substantial number of patients either do not respond adequately to these drugs or are unable to tolerate their adverse effects.^[7,8]^ Therefore, effective and safe alternative therapeutic options to manage PSD are essential.

Chinese herbal medicine (CHM), as a part of traditional Chinese medicine therapy, is a complementary and alternative treatment system that is being widely used in the world.^[9]^ Wendan decoction (WDD) is a clinically widely used prescription for PSD, which consists of Banxia (Pinellia ternata), Zhuru (Bamboo shavings), Bai FuLing (Poria), Zhishi (Immature Trifoliate-orange fruit), Shengjiang (Ginger), Chenpi (Tangerine peel), and Gancao (Licorice root).^[10]^ Although WDD has been suggested to be effective in the treatment of PSD,^[11,12]^ no previous meta-analysis has provided evidence to determine whether WDD administration is an ideal method to treat PSD. Therefore, we aim to conduct a meta-analysis to evaluate the effectiveness and safety of WDD in the treatment of PSD.

## 2 Methods

### 2.1 Study registration

This protocol has been prepared in accordance with the statement guidelines of the preferred reporting items for systematic reviews and meta-analysis protocols (PRISMA-P).^[13]^ This study was prospectively registered in the International Platform of Registered Systematic Review and Meta-Analysis Protocols (registration number INPLASY2021110018) on November 06, 2021.

### 2.2 Eligibility criteria

#### 2.2.1 Types of studies

Randomized controlled trials (RCTs) of WDD for the management of patients with PSD will be included. Only studies in Chinese or English will be included. Animal studies will be excluded.

#### 2.2.2 Types of participants

Patients clinically diagnosed with PSD will be included, regardless of race, sex, age, disease course, and severity.

#### 2.2.3 Types of interventions

The control group will include patients treated with antidepressants. Patients in the experimental group will receive WDD or treatment adjustment based on WDD alone or in combination with antidepressants.

#### 2.2.4 Outcome measures

##### 2.2.4.1 Primary outcomes

The primary outcomes will be the Hamilton depression scale (HAMD) score, including the HAMD 17-item score and HAMD 24-item score, and the total effective rate. As mentioned earlier, a reduction of ≥25% in the HAMD score will be used to indicate that the treatment is effective.^[14]^

##### 2.2.4.2 Secondary outcomes

The secondary outcomes will include the National Institutes of Health Stroke Scale (NIHSS), Degree of neurological impairment, Barthel Index (BI), and incidence of adverse events.

### 2.3 Search strategy

Seven electronic databases will be searched: the Cochrane Library, PubMed, EMBASE, China National Knowledge Infrastructure, Wan Fang databases, Chinese Biomedical Literature Database, and China Science and Technology Journal Database. Each database will be searched from its inception to November 2021. The details of the retrieval strategy in PubMed are summarized in Table 1. Similar retrieval strategies will be followed for each electronic database.

**Table 1:** Search strategy for the PubMed database.

Number	Search terms
#1	“Wendan decoction”[Mesh] OR “Wendan tang ”[Mesh]
#2	“Wendan decoction”[Ti/Ab] OR “Wendan tang ”[Ti/Ab]
#3	#1or#2
#4	“Post-stroke depression”[Mesh] OR “depression after stroke”[Mesh]
#5	“Post-stroke depression”[Ti/Ab] OR “depression after stroke”[Ti/Ab]
#6	#4or#5
#7	Randomized controlled trial” [MeSH] or “controlled clinical trial” [MeSH]
#8	Randomized controlled trial” [Ti/Ab] or “randomized” [Ti/Ab] or “clinical trial” [Ti/Ab]
#9	#7or#8
10	#3and#6and #9

### 2.4 Study selection

**Figure 1:**
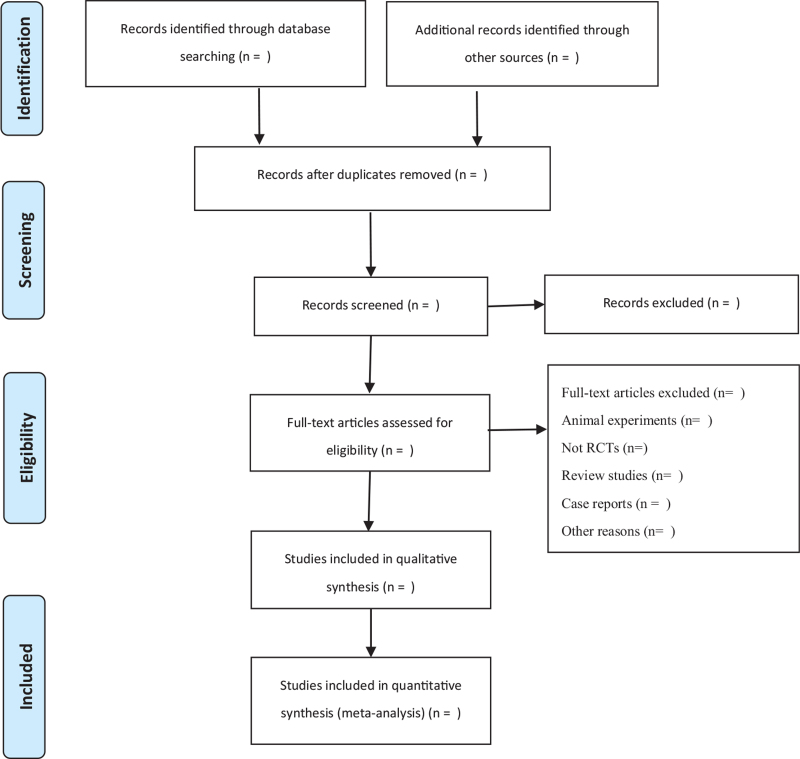
PRISMA flow chart.

The retrieved articles will be input into Noteexpress software 3.2 to count and eliminate duplicates. The 2 researchers will read the titles and abstracts independently to eliminate irrelevant articles in accordance with the inclusion and exclusion criteria, and then further screen the articles by reading the remaining full text. Disagreements during the study selection process will be resolved through discussion with the third researcher. The PRISMA flowchart (Fig. 1) shows the details of the selection process.

### 2.5 Data extraction and management

Two independent researchers will extract data independently through a data extraction table. The data extraction table contains basic information, study characteristics and methodology, participant characteristics, details of interventions and comparators, and information regarding outcomes. Disagreements during the data extraction process will be resolved through discussion with the third researcher. If the data provided in the included articles are insufficient and unclear, the corresponding author will be contacted for more information.

### 2.6 Risk of bias assessment

The 2 researchers will independently assess the risk of bias of the included RCTs by using the Cochrane risk of bias assessment tool (RevMan v5.4). This tool covers 7 domains: random sequence generation, allocation concealment, blinding of participants and personnel, blinding of outcome assessment, incomplete outcome data, selective reporting, and other biases. The 7 items will be judged as showing low, unclear, or high risk of bias. Any discrepancies will be resolved through consultation with a third researcher.

### 2.7 Data synthesis and analysis

#### 2.7.1 Data synthesis

If the included data are suitable, a meta-analysis will be conducted using RevMan v5.4 software (Cochrane Community, London, UK). If not, systematic narrative synthesis or qualitative research will be performed to explain the findings of the included studies.

#### 2.7.2 Measurement of the treatment effect

Mean differences and 95% CIs will be used for continuous variables and risk ratios (RRs) and 95% CIs will be used for dichotomous variables.

#### 2.7.3 Assessment of heterogeneity

Heterogeneity will be identified by the Q test and *I*^2^ statistic. If *P* > .1 and *I*^2^ < 50%, the heterogeneity in the studies might not be important, and we will adopt a fixed-effect model. If *P* < .1% or *I*^2^ > 50%, the inconsistency across studies may be substantial, so we will adopt a random-effects model after excluding the clinical heterogeneity.

#### 2.7.4 Subgroup analysis

If the heterogeneity of the included data is significant, we will conduct subgroup analyses according to the course of treatment, differences in severity, intervention measures for the control group, etc.

#### 2.7.5 Sensitivity analysis

To test the robustness and reliability of the meta-analysis, we will conduct sensitivity analysis using the following aspects:

(1) Eliminating low-quality studies
(2) Replacing random-effects models with fixed-effects models, and vice versa.

#### 2.7.6 Reporting bias

Funnel plots will be used to assess the publication bias if sufficient studies (≥10 studies) are included. Alternatively, the Egger test will be conducted with STATA 13.0 Software.

#### 2.7.7 Grading the quality of evidence

We will use The Grading of Recommendations Assessment, Development, and Evaluation ^[15]^ to evaluate the quality of evidence for the outcomes. Levels of evidence will be ranked as “high,” “moderate,” “low,” and “very low.”

## 3 Discussion

CHM is commonly used as an alternative supplement therapy, and it has been shown to be beneficial for PSD. WDD is one of the commonly used CHM prescriptions for PSD.^[16]^ Although previous randomized controlled studies have confirmed the efficacy of WDD in the treatment of PSD,^[17,18]^ comprehensive systematic evaluations of the efficacy of WDD in the treatment of PSD are rare. Therefore, the purpose of this meta-analysis was to evaluate the efficacy and safety of WDD in the treatment of PSD. The findings of this meta-analysis will provide evidence-based data for the application of WDD in the treatment of PSD.

## Acknowledgments

The authors thank Dr Wen Zehuai for his suggestions and comments on the manuscript.

## Author contributions

Conceptualization: Wenbin Fu, Yonghui Hou

Data collection: Yonghui Hou, Wenwen Pang

Formal analysis: Yonghui Hou, Wenwen Pang

Funding acquisition: Jing Gao,Wenbin Fu,

Software: Yonghui Hou

Supervision: Wei Si

Drafting the manuscript: Yonghui Hou

Reviewing and editing the manuscript: Yonghui Hou, Baile Ning

**Conceptualization:** Yonghui Hou, Wenbin Fu.

**Data curation:** Yonghui Hou, Wenen Pang.

**Formal analysis:** Yonghui Hou, Wenen Pang.

**Funding acquisition:** Jing Gao, Wenbin Fu.

**Methodology:** Yonghui Hou.

**Project administration:** Jing Gao.

**Supervision:** Wei Si.

**Writing – original draft:** Yonghui Hou.

**Writing – review & editing:** Yonghui Hou, Baile Ning.
